# Phosphorylation of TET Proteins Is Regulated via *O*-GlcNAcylation by the *O*-Linked *N*-Acetylglucosamine Transferase (OGT)*[Fn FN2]

**DOI:** 10.1074/jbc.M114.605881

**Published:** 2015-01-07

**Authors:** Christina Bauer, Klaus Göbel, Nagarjuna Nagaraj, Christian Colantuoni, Mengxi Wang, Udo Müller, Elisabeth Kremmer, Andrea Rottach, Heinrich Leonhardt

**Affiliations:** From the ‡ Biocenter, Ludwig-Maximilians University Munich, 82152 Planegg-Martinsried,; the § Max Planck Institute of Biochemistry, D-82152 Martinsried,; the ¶ Institute for Molecular Immunology, Helmholtz Center Munich, 81377 München-Groβhadern, and; the ‖ Center for Integrated Protein Science Munich (CIPSM), 81377 München, Germany

**Keywords:** 5-Hydroxymethylcytosine (5-hmC), Dioxygenase, Epigenetics, O-Linked N-Acetylglucosamine (O-GlcNAc), Phosphorylation, Post-translational Modification (PTM), OGT, TET Proteins

## Abstract

TET proteins oxidize 5-methylcytosine to 5-hydroxymethylcytosine, 5-formylcytosine, and 5-carboxylcytosine and thus provide a possible means for active DNA demethylation in mammals. Although their catalytic mechanism is well characterized and the catalytic dioxygenase domain is highly conserved, the function of the regulatory regions (the N terminus and the low-complexity insert between the two parts of the dioxygenase domains) is only poorly understood. Here, we demonstrate that TET proteins are subject to a variety of post-translational modifications that mostly occur at these regulatory regions. We mapped TET modification sites at amino acid resolution and show for the first time that TET1, TET2, and TET3 are highly phosphorylated. The *O*-linked GlcNAc transferase, which we identified as a strong interactor with all three TET proteins, catalyzes the addition of a GlcNAc group to serine and threonine residues of TET proteins and thereby decreases both the number of phosphorylation sites and site occupancy. Interestingly, the different TET proteins display unique post-translational modification patterns, and some modifications occur in distinct combinations. In summary, our results provide a novel potential mechanism for TET protein regulation based on a dynamic interplay of phosphorylation and *O*-GlcNAcylation at the N terminus and the low-complexity insert region. Our data suggest strong cross-talk between the modification sites that could allow rapid adaption of TET protein localization, activity, or targeting due to changing environmental conditions as well as in response to external stimuli.

## Teaser

**Background:** TET proteins oxidize 5-methylcytosine and contribute to active DNA demethylation.

**Results:** *O*-Linked GlcNAc transferase modifies TET proteins with GlcNAc and thereby reduces TET phosphorylation.

**Conclusion:** TET proteins are subjected to a dynamic interplay of post-translational modifications at low-complexity regions.

**Significance:** This first map of TET phosphorylation and *O*-GlcNAcylation sites at amino acid resolution provides a valuable resource for future studies of TET regulation.

## Introduction

A major epigenetic mechanism of gene regulation in higher eukaryotes is methylation of DNA at C5 of cytosines (1, 2). Recently, the family of TET (ten-eleven translocation) proteins has been shown to successively oxidize 5-methylcytosine to 5-hydroxymethylcytosine, 5-formylcytosine, and 5-carboxylcytosine (3–6), providing novel insights into the dynamics of DNA modifications. TET proteins are also active on genomic thymine residues, leading to the generation of 5-hydroxyuracil (7). In Gnathostomata, there are three TET proteins, TET1, TET2, and TET3 (8), which show distinct expression patterns and functions in different tissues or during development (9–13). TET1 and TET2 are highly expressed in mouse embryonic stem cells (mESCs)^4^ and are associated with oxidation of transcription start sites and gene bodies, respectively (14). TET3 is up-regulated in the oocyte and oxidizes the silenced paternal pronuclear DNA (10, 15). High levels of TET proteins and genomic 5-hydroxymethylcytosine are described for neuronal tissues (11, 16–18). In several patients with myeloid malignancies, mutations of *TET2* correlate with decreased 5-hydroxymethylcytosine levels and altered gene expression patterns (19–22).

The activity of TET proteins directly depends on two cofactors: Fe(II) and 2-oxoglutarate (3, 8). Interestingly, gain-of-function mutations of the enzymes responsible for 2-oxoglutarate synthesis, IDH1 and IDH2, have been associated with tumorigenesis, in particular glioblastomata and acute myeloid leukemia (20, 23, 24). These mutations lead to the synthesis of 2-hydroxyglutarate, a potent inhibitor of 2-oxoglutarate-dependent dioxygenases such as TET proteins (24, 25). Because IDH1 and IDH2 are enzymes of the Krebs cycle, these findings represent a direct link of TET protein activity to metabolism, especially because low 5-hydroxymethylcytosine levels are found in acute myeloid leukemia patients not only with *TET2* loss-of-function mutations but also with *IDH2* gain-of-function mutations (20). Besides 2-hydroxyglutarate, ascorbate has also been shown to influence cytosine oxidation by TET proteins (26–28). In summary, TET protein activity appears to be modulated by several small molecules, either inhibitory such as 2-hydroxyglutarate or stimulating such as ascorbate.

TET proteins are influenced not only by certain metabolites but also by interacting proteins. TET1 forms complexes with heterochromatin-associated proteins such as HDAC1, HDAC2, SIN3A, and EZH2 (29). All three TET proteins interact with a variety of factors of the base-excision repair pathway, including PARP1, LIG3, and XRCC1, and also with several DNA glycosylases, including thymine-DNA glycosylase, NEIL1, and MDB4 (30). Another known interactor of TET proteins is the glycosyltransferase OGT (31–36), which represents an additional interesting connection with metabolism. OGT catalyzes the addition of a GlcNAc group to serine or threonine residues of target proteins (37). Its activity is dependent on the availability of a variety of metabolic molecules such as glucose, ATP, glutamine, and acetyl-CoA (38). The association of OGT with TET proteins has been reported to influence histone modifications and gene expression (31, 36), TET1 protein stability (33) and activity (34), and TET3 subcellular localization (35).

TET protein activity is widely studied in the context of development, tumorigenesis, and metabolic conditions. However, only very little is known about the structure and function of the non-catalytic domains of TET proteins. In this study, we show that TET proteins are subject to a large number of post-translational modifications (PTMs), predominantly occurring at the two low-complexity regions, which display only little sequence conservation: the N terminus and the insert region that separates the two parts of the catalytic dioxygenase domain and is predicted to be unstructured (8). We demonstrate that TET proteins are phosphorylated and that this phosphorylation can be suppressed via *O*-GlcNAcylation by the glycosyltransferase OGT. Detailed mapping of modification sites to the protein sequence shows that mostly the N terminus and insert region of TET proteins are subjected to PTMs and that their regulation depends on a dynamic interplay of different PTMs.

## EXPERIMENTAL PROCEDURES

### 

#### 

##### Antibody Generation

**FIGURE 1.**
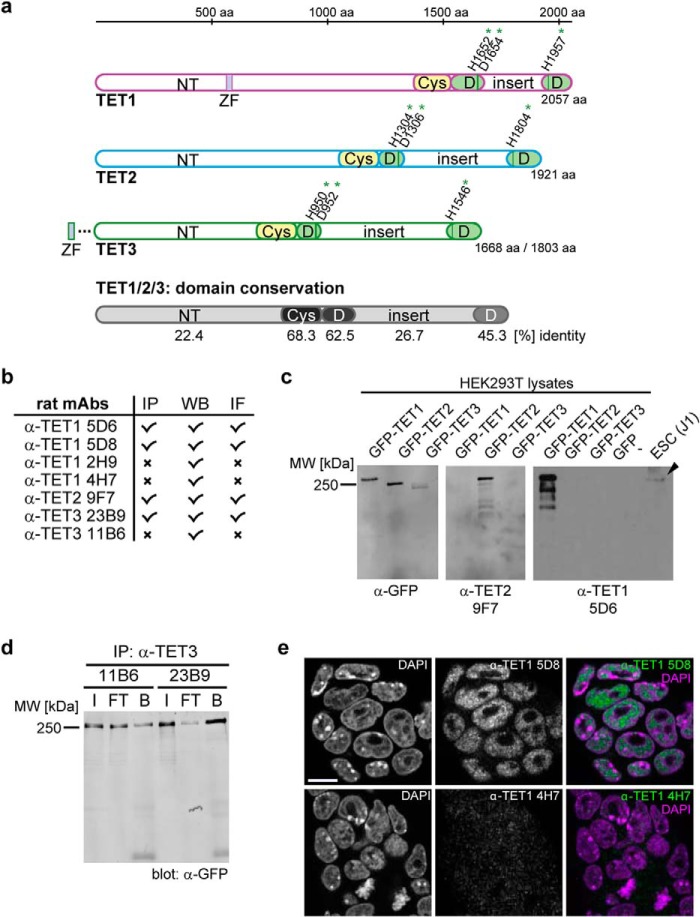
**Generation of anti-TET monoclonal antibodies.** *a*, schematic representation of the domain architecture of the three murine TET proteins. The catalytic dioxygenase domain (*D*) is split in two parts, separated by a presumably unstructured low-complexity insert (8), and is N-terminally preceded by a cysteine-rich region (*Cys*). The Fe(II)-binding residues are marked with *green asterisks*. The N terminus (*NT*) of TET1 contains a C*XXC*-type zinc finger (*ZF*). TET3 exists in two isoforms, one with a zinc finger and one without (41). The mean percent identity of the single domains of TET1, TET2, and TET3 is represented by different shades of *gray* and was calculated with Clustal 2.1 (59). *aa*, amino acids. *b*, overview of the generated anti-TET monoclonal antibodies (*mAbs*) and their possible applications. *IP*, immunoprecipitation; *WB*, Western blotting; *IF*, immunofluorescence; *x*, antibody not suited for the indicated application. *c*, example of Western blot analysis of two anti-TET antibodies with an anti-GFP antibody as a positive control. The antibodies detected only their target protein, but not the other two TET proteins. The WT protein from mESC whole cell lysates was also detected specifically (*black arrowhead*). *d*, example of an immunoprecipitation experiment with the indicated anti-TET3 antibodies. Clone 23B9 efficiently precipitated TET3 compared with clone 11B6. Western blot analysis was performed with an anti-GFP antibody. *I*, input; *FT*, flow-through; *B*, bound). *e*, immunofluorescence staining of mESCs with anti-TET1 antibodies (clones 5D8 and 4H7) and DAPI as a DNA counterstain. Whereas clone 5D8 showed a clear nuclear pattern, clone 4H7 displayed only a weak and diffuse signal. Confocal imaging was performed with a Leica TCS SP5 confocal laser scanning microscope with a ×63/1.4 numerical aperture Plan-Apochromat oil immersion objective. *Scale bar* = 5 μm.

A His-tagged protein fragment from the insert region of each TET protein (see Fig. 1*a*) was expressed in *Escherichia coli* BL21(DE3) cells (Novagen, Darmstadt, Germany) and purified with the TALON Superflow metal affinity resin system (Clontech, Saint Germain, France) under native conditions as described previously (39). Amino acids 1682–1914 for TET1, amino acids 1332–1779 for TET2, and amino acids 976–1521 for TET3 were used as antigens. Approximately 100 μg of each antigen was injected both intraperitoneally and subcutaneously into Lou/C rats using CPG2006 (TIB MOLBIOL, Berlin, Germany) as adjuvant. After 8 weeks, the immune response was boosted intraperitoneally and subcutaneously 3 days before fusion. Fusion of the myeloma cell line P3X63-Ag8.653 with rat immune spleen cells was performed using PEG 1500 (Roche Diagnostics Deutschland GmbH, Mannheim, Germany). After fusion, the cells were cultured in 96-well plates using RPMI 1640 medium with 20% fetal calf serum, penicillin/streptomycin, pyruvate, and nonessential amino acids (PAA, Linz, Austria) supplemented with aminopterin (Sigma). Hybridoma supernatants were tested in a solid-phase immunoassay. Microtiter plates were coated overnight with His-tagged TET antigens at a concentration of 3–5 μg/ml in 0.1 m sodium carbonate buffer (pH 9.6). After blocking with nonfat milk (Frema Reform, granoVita, Heimertingen, Germany), hybridoma supernatants were added. Bound rat monoclonal antibodies were detected with a mixture of biotinylated mouse monoclonal antibodies against rat IgG heavy chains, avoiding anti-IgM monoclonal antibodies (anti-IgG1, anti-IgG2a, and anti-IgG2b (American Type Culture Collection, Manassas, VA) and anti-IgG2c (Ascenion GmbH, Munich, Germany)). The biotinylated monoclonal antibodies were visualized with peroxidase-labeled avidin (Alexis, San Diego, CA) and *o*-phenylenediamine as chromogen in the peroxidase reaction. Anti-TET1 (clones 5D6, 5D8, 2H9, and 4H7; rat IgG2a), anti-TET2 (clone 9F7; rat IgG2a), and anti-TET3 (clones 11B6 and 23B9; rat IgG2a) antibodies were stably subcloned and further characterized (see Fig. 1*b*).

##### mESC Culture, Co-immunoprecipitation, and MS/MS Analysis

mESCs (J1) were cultured as described previously (9). Endogenous TET1 and TET2 proteins were pulled out via monoclonal antibodies (clones 5D6, 5D8, and 9F7) coupled to protein G-Sepharose beads as described (39). After co-immunoprecipitation, protein samples were digested on beads with trypsin according to standard protocols. Peptide mixtures were analyzed by electrospray MS/MS spectrometry. Experiments were performed with an LTQ Orbitrap XL mass spectrometer (Thermo Scientific, Waltham, MA). Spectra were analyzed with Mascot software (Matrix Science, Boston, MA).

##### Expression Constructs

Expression constructs for GFP-TET1, GFP-TET2, GFP-TET3, GFP, and Cherry were described previously (40–42). To generate the Cherry-OGT construct, the coding sequence was amplified using cDNA from E14 mESCs as template and subcloned into the pCAG-Cherry-IB vector. Expression constructs for Cherry-OGT(H508A) (hereafter referred to as OGT^mut^) were generated by overlap extension PCR. All constructs were verified by DNA sequencing (Eurofins Genomics, Ebersberg, Germany).

##### HEK293T Culture, Co-immunoprecipitation, and Western Blot Analysis

Co-immunoprecipitation followed by Western blotting with GFP- and Cherry-tagged proteins expressed in HEK293T cells was performed as described previously (30). *O*-GlcNAc was detected with a mouse monoclonal antibody (RL2, Abcam, Cambridge, United Kingdom) and an Alexa 647N-conjugated secondary antibody (Sigma).

##### Sample Preparation for Mass Spectrometric Analysis

All experiments were performed in biological triplicates. GFP-tagged TET proteins and/or Cherry-tagged OGT and OGT^mut^ were expressed in HEK293T cells. Cell lysis with radioimmune precipitation assay buffer and immunoprecipitation with the GFP-Trap (ChromoTek GmbH, Martinsried, Germany) were performed as described previously (30). After immunoprecipitation, samples on beads were rinsed two times with wash buffer (20 mm Tris-HCl (pH 7.5), 300 mm NaCl, and 0.5 mm EDTA) and two times with immunoprecipitation buffer (20 mm Tris-HCl (pH 7.5), 150 mm NaCl, and 0.5 mm EDTA).

100 μl of denaturation buffer (6 m guanidine hydrochloride, 10 mm tris(2-carboxyethyl)phosphine, and 40 mm chloroacetamide in 100 m Tris (pH 8.5)) was added to the beads and heated at 70 °C for 5 min. The samples were then subjected to sonication in a Diagenode Bioruptor Plus system (UCD-300-TO) at maximum power settings for 10 cycles consisting of a 30-s pulse and 30-s pause. Following sonication, the samples were diluted 1:10 with digestion buffer (25 mm Tris (pH 8.5) containing 10% acetonitrile) and mixed by vortexing prior to enzyme digestion. Each sample was digested with 1 μg of endoproteinase Lys-C (Wako Chemicals, Neuss, Germany) for 4 h with subsequent digestion using 1 μg of trypsin (Promega, Madison, WI) under gentle rotation at 37 °C. After digestion, the samples were placed in a SpeedVac concentrator for 10 min to remove acetonitrile from the sample before StageTip purification using SDB-XC material (43). Peptides were then eluted from the StageTip and placed in the SpeedVac concentrator to reduce the sample volume to ∼6 μl, and 5 μl of the sample was injected onto the column for MS/MS analysis.

##### LC-MS/MS and Data Analysis

Samples were loaded onto a column (15-cm length and 75-μm inner diameter; New Objective, Woburn, MA) packed with 3-μm ReproSil C_18_ beads (Dr. Maisch GmbH, Ammerbuch-Entringen, Germany) using an EASY-nLC autosampler (Thermo Scientific) coupled via a nanoelectrospray source to the LTQ Orbitrap XL mass spectrometer. Each sample was analyzed using a 2-h reversed-phase gradient and a top 5 method for data-dependent acquisition. Full scans were acquired in the Orbitrap mass spectrometer after accumulating up to 1 × 10^6^ charges, and MS/MS with the five most abundant precursors was performed using low-energy ion-trap collision-induced dissociation. MS/MS spectra were recorded using the ion trap by radial ejection.

All raw files were analyzed using the MaxQuant computational proteomics platform (version 1.4.1.6) (44). Peak lists were searched with an initial mass deviation of 7 ppm and fragment ion deviation of 0.5 Thomson. Carbamidomethylation was used as a fixed modification. Oxidation of methionine; phosphorylation of serine, threonine, and tyrosine; *O*-GlcNAcylation of serine and threonine; ubiquitination (diglycine motif) of lysine; and acetylation of the protein N terminus were used as variable modifications. All unmodified and oxidized methionine- and *N*-acetylation-containing peptides were used for protein quantification. The MaxQuant software quantifies the different versions of modified peptides in a label-free fashion. Briefly, the occupancy reflects the extracted signal differences between modified and unmodified peptides and also includes the protein ratios between samples. The different forms of modified peptides, *e.g.* peptides with single, double, and triple *O*-GlcNAc sites, are individually quantified and listed separately in the output (supplemental Table S4). Details on label-free quantification of modification sites are provided elsewhere (45).

MaxQuant output data were further analyzed with Perseus software (version 1.5.0.15) (44). Only modifications that were detected in at least two of three biological replicates in at least one experimental setup were included in the analysis. PTMs that were detected in non-unique peptides were also excluded. Significance was tested using a Student's two-tailed paired *t* test.

## RESULTS

### 

#### 

##### Characterization of Anti-TET Antibodies

The three TET proteins share a common domain architecture: the C-terminal catalytic dioxygenase domain is split into two parts separated by a low-complexity insert region and is preceded by an extension enriched in cysteines (8). All three TET proteins have a large N-terminal region that is mostly uncharacterized so far, except for a C*XX*C-type zinc finger at the N terminus of TET1 and TET3 (8, 40, 47). Murine TET3 exists in two isoforms: one with the zinc finger and one without (41). The cysteine-rich region and the split dioxygenase domain are conserved among the three murine TET proteins, whereas the N terminus and insert region display only little sequence similarity (Fig. 1*a*). The three-dimensional structure of mammalian TET proteins remains unresolved, with the exception of the cysteine-rich and dioxygenase domains of TET2 (48), leaving the structure and function of the N terminus and low-complexity insert unknown.

We generated antibodies against murine TET1, TET2, and TET3 using protein fragments derived from the insert region of the catalytic domains as antigens. The rat monoclonal antibodies were tested for their applicability in Western blotting, immunoprecipitation, and immunofluorescence (Fig. 1*b*). Fig. 1 (*c–e*) shows exemplary data from the antibody characterization process of selected clones. For antibody testing, GFP-tagged TET proteins were expressed in HEK293T cells and detected in the cell lysate by Western blotting using anti-TET antibodies and an anti-GFP antibody as a positive control (Fig. 1*c* and data not shown). For immunoprecipitation, antibodies were coupled to protein G beads, incubated with the cell lysates, and analyzed by anti-GFP Western blotting for efficient pulldown of the respective TET protein (Fig. 1*d* and data not shown). mESCs were used to test the suitability of the obtained antibodies for immunofluorescence. Antibodies preselected for specificity in Western blot analyses that showed a clear nuclear staining were judged as applicable in immunofluorescence (Fig. 1*e* and data not shown).

##### TET Proteins Interact with and Are O-GlcNAcylated by OGT

**FIGURE 2.**
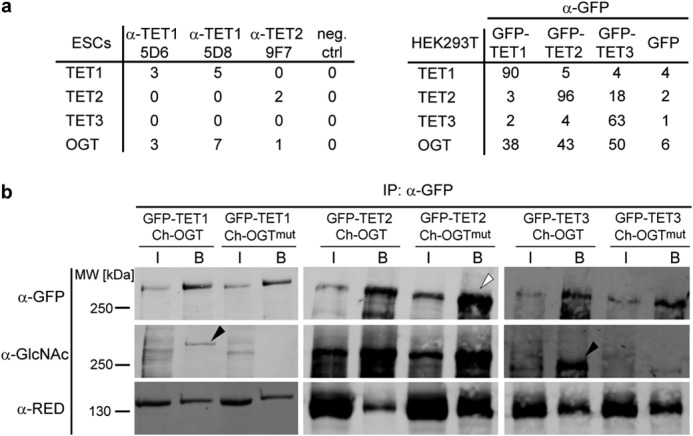
**TET proteins interact with OGT and are *O*-GlcNAcylated.** *a*, number of unique peptides detected in immunoprecipitation experiments followed by LC-MS/MS. *Left*, immunoprecipitation of endogenous TET1 or TET2 with the indicated antibodies. Protein G beads without antibody were used as a negative control (*neg. ctrl*). *Right*, immunoprecipitation of GFP-tagged TET1, TET2, or TET3 expressed in HEK293T cells. Pulldown of GFP served as a negative control. *b*, Western blot analysis of TET1, TET2, and TET3 specifically enriched with GFP-Trap. Upon coexpression of active OGT, the *O*-GlcNAcylation signal increased for TET1 and TET3 (*black arrowheads*) compared with coexpression of catalytically inactive OGT^mut^. For TET2, protein levels in the OGT^mut^ samples were higher (*white arrowhead*), whereas the *O*-GlcNAc signal remained constant, suggesting a higher proportion of *O*-GlcNAcylated TET2 in the OGT sample. Interaction between TET proteins and OGT was independent of OGT activity. Anti-RED antibody (60) detected the coexpressed Cherry (*Ch*)-tagged OGT. *IP*, immunoprecipitation; *I*, input; *B*, bound.

As a first step toward understanding the regulation of TET proteins, we screened for interaction partners in mESCs. Because TET1 and TET2 are constitutively expressed in mESCs, clones 5D6 (anti-TET1), 5D8 (anti-TET1), and 9F7 (anti-TET2) were used to pull down endogenous TET1 and TET2. Subsequent LC-MS/MS analysis revealed that both TET1 and TET2 interacted with the glycosyltransferase OGT. In accordance with this result, co-immunoprecipitation analysis of GFP-tagged TET1, TET2, and TET3 expressed in HEK293T cells shows high enrichment of OGT in the pulldown (Fig. 2*a*).

Having observed the interaction between TET proteins and OGT, we examined whether TET proteins are modified by OGT and screened for *O*-GlcNAcylation, the modification that is transferred to the OH group of serine or threonine residues of target proteins by OGT (38, 49). To this end, we specifically enriched GFP-tagged TET proteins coexpressed with either OGT or its catalytically inactive point mutant OGT^mut^ with GFP-Trap and probed the subsequent Western blot with an anti-GlcNAc antibody. All three TET proteins were found to be increasingly *O-*GlcNAcylated upon the coexpression of catalytically active OGT (Fig. 2*b*).

##### O-GlcNAcylation Reduces Phosphorylation of TET Proteins

**FIGURE 3.**
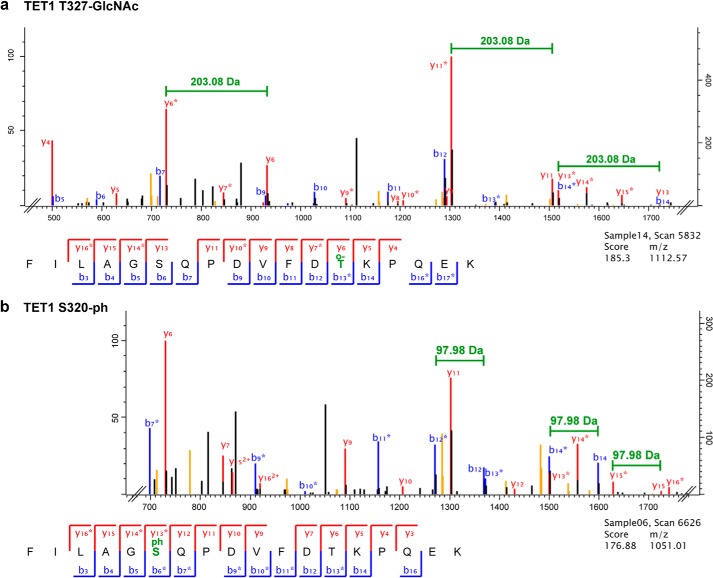
**Exemplary MS/MS spectra of modified TET1 peptides.** *a*, MS/MS spectrum of a TET1 peptide modified with *O*-GlcNAc (*o-*) at the threonine residue. *O*-GlcNAcylation is characterized by a neutral loss of 203.8 Da as indicated. y ions are depicted in *red*, and b ions are depicted in *blue*. Labeling of neutral losses of H_2_O or NH_3_ (*orange peaks*) has been removed for clarity. Fully annotated spectra are provided in supplemental Data S2 and S3. *b*, MS/MS spectrum of the same TET1 peptide phosphorylated (*-ph*) at the serine residue. Phosphorylated ions show a neutral loss of 97.98 Da as indicated. y ions are depicted in *red*, b ions are depicted in *blue*. Labeling of neutral losses of H_2_O or NH_3_ (*orange peaks*) has been removed for clarity. Fully annotated spectra are provided in supplemental Data S2 and S3.

**FIGURE 4.**
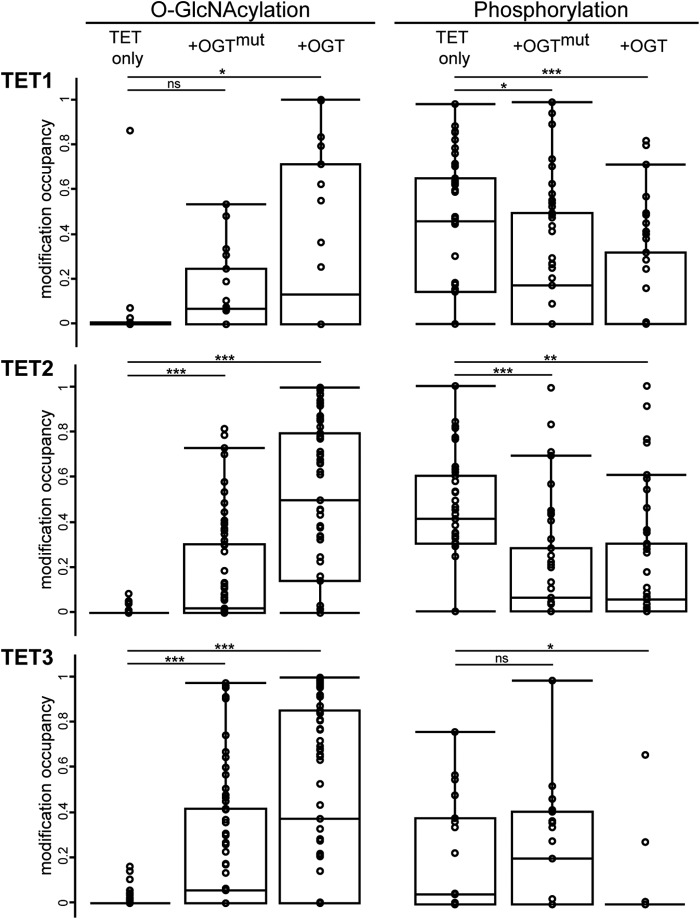
**TET phosphorylation is reduced upon *O*-GlcNAcylation.** Box plots depict the distribution of *O*-GlcNAc and phosphorylation occupancy in the three conditions: expression of TET protein only, coexpression of OGT^mut^, and coexpression of OGT. Missing values have been substituted with an occupancy of 0.005, with 0.001 being the lowest measured occupancy. Mean occupancies of single sites are provided in Tables 1–3. *, *p* < 0.05 (Student's *t* test); **, *p* < 0.01; ***, *p* < 0.001; *ns*, not significant.

To identify OGT-dependent *O*-GlcNAcylation sites on TET proteins, we performed mass spectrometric analysis of semipurified proteins. We therefore expressed GFP-tagged TET1, TET2, and TET3 in HEK293T cells with either OGT or OGT^mut^ or without interactor. After pulldown with GFP-Trap and stringent washing steps, the samples were analyzed by LC-MS/MS. An overall sequence coverage of ∼50% was achieved for TET1, ∼60% for TET2, and ∼65% for TET3 (supplemental Data S1 and Table S4). For data analysis, only sites were considered that were detected in at least two of three biological replicates. Fig. 3*a* shows an exemplary MS/MS spectrum of an *O*-GlcNAcylated TET1 peptide. Without coexpression of interactor, only a few residues on TET proteins were found to be *O*-GlcNAcylated at low site occupancy. Coexpression of OGT led to a strong increase in both the number of *O*-GlcNAcylation sites and site occupancy for TET2 and TET3. The difference in the number of *O*-GlcNAc sites was either due to *de novo* modification by OGT or because the site occupancy without OGT coexpression was below the detection limit. For TET1, however, the *O*-GlcNAc pattern was relatively heterogeneous, and only a few *O*-GlcNAc sites could be detected. This heterogeneity is also illustrated by the fact that residues 1327 and 327, which were *O*-GlcNAcylated in the TET1 samples, were modified only in one of three replicates in the TET1/OGT samples. Although Cherry-OGT^mut^ is supposed to be catalytically inactive, coexpression led to a small increase in *O*-GlcNAcylation and represented a distinct state from basal levels (Fig. 4 and Tables 1–3).

**TABLE 1:** **Detected modified peptides of TET1** (ph), phosphorylated; (*o*), *O*-GlcNAcylated; (ox), oxidized. Localization probability was calculated with MaxQuant software (44). Residue numbering refers to the murine protein sequences specified in supplemental Data S1. The arithmetic mean ± S.D. of the occupancy is depicted for each data set. ND, not detected.

Modified amino acid	Localization probability	Modified sequence	Mean TET1	Mean TET1 + OGT^mut^	Mean TET1 + OGT
160	1.00	H … ATVS(ph)PGTENGEQNR	0.15 ± ND	0.98 ± ND	0.34 ± 0.08
177	1.00	CLVEGES(ph)QEITQSCPVFEER	0.63 ± 0.01	0.32 ± 0.15	0.48 ± ND
253	0.85	NT(*o*)SNQLADLSSQVESIK	ND	0.07 ± 0.01	0.37 ± ND
270	0.84	LS(*o*)DPSPNPTGSDHNGFPDSSFR	ND	0.08 ± 0.02	0.67 ± 0.06
320	1.00	FILAGS(ph)QPDVFDTKPQEK	0.60 ± 0.16	0.44 ± 0.03	0.30 ± 0.20
327	1.00	FILAGSQPDVFDT(*o*)KPQEK*^a^*	0.32 ± 0.47	0.36 ± 0.15	0.55 ± ND
556	0.81	A … STSS(ph)PPCNSTPPMVER	0.23 ± 0.10	ND	ND
561	0.89	A … STSSPPCNS(ph)TPPM(ox)VER	0.20 ± 0.09	0.88 ± ND	ND
734	0.98	QQTNPS(ph)PTFAQTIR	0.44 ± ND	0.46 ± 0.33	0.32 ± ND
736	0.96	QQTNPSPT(ph)FAQTIR	0.67 ± 0.06	ND	ND
794	0.77	DAM(ox)SVTTS(*o*)GGECDHLK	ND	0.48 ± ND	1.00 ± 0.00
854	1.00	DGS(ph)PVQPSLLSLMK	0.73 ± 0.13	0.54 ± 0.07	0.25 ± 0.34
892	0.70	L … SESSS(ph)PSKPEK	0.51 ± 0.48	0.27 ± 0.03	0.79 ± ND
950	1.00	S(ph)PDSFATNQALIK*^b^*	0.68 ± 0.26	0.72 ± 0.20	0.49 ± 0.11
969	0.74	SQGYPSS(ph)PT …	0.61 ± 0.03	ND	ND
1327	0.66	REAQT(*o*)SSN … K*^a^*	0.01 ± 0.00	ND	0.79 ± ND
1964	0.89	ELHATTSLRS(ph)PK	0.33 ± 0.21	0.17 ± ND	0.47 ± 0.33
2016	1.00	PADRECPDVS(ph)PEANLSHQIPSR	0.68 ± 0.21	0.37 ± 0.18	0.81 ± ND
2016	0.56	PADRECPDVS(*o*)PEANLSHQIPSR	ND	0.26 ± 0.10	0.55 ± 0.41
2042	0.99	DNVVTVS(ph)PYSLTHVAGPYNR	0.73 ± 0.12	ND	0.38 ± ND

*^a^* Basal *O*-GlcNAc sites.

*^b^* Persistent phosphorylation sites.

Because *O*-GlcNAcylation occurs at serine or threonine residues of the target protein, we also screened for another PTM that can occur at these amino acids, namely phosphorylation. Interestingly, high phosphorylation of TET1, TET2, and, to a lesser extent, TET3 was observed. Phosphorylation of all TET proteins decreased significantly upon coexpression of OGT regarding both site occupancy and the number of detected phosphorylation sites (Fig. 4 and Tables 1–3). An example of a MS/MS spectrum of a phosphorylated TET1 peptide is shown in Fig. 3*b*. The MS/MS spectra of all modified TET peptides are provided in supplemental Data S2 and S3.

##### PTMs Occur Mostly at the N terminus and in the Low-complexity Insert of TET Proteins

**FIGURE 5.**
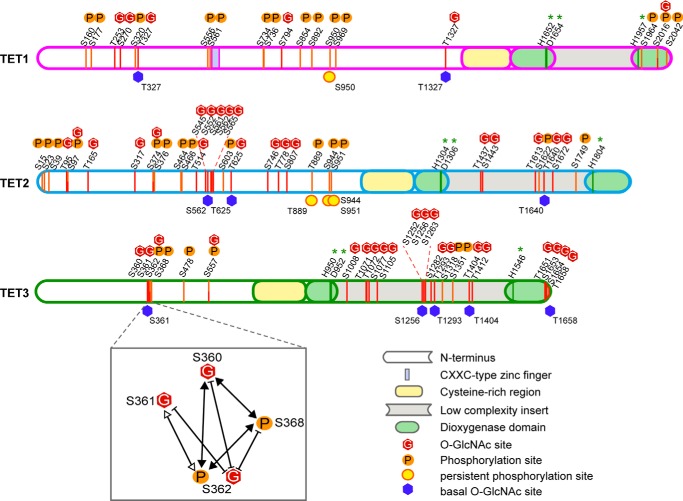
**N termini and insert regions of TET proteins are densely modified.** Shown are a schematic and scaled mapping of all TET phosphorylation and *O*-GlcNAcylation sites in the protein sequence. Modifications are found mostly in the N terminus and insert region and rarely occur at the same residue. Residue numbering refers to the murine protein sequences specified in supplemental Data S1. *Green asterisks* indicate catalytic Fe(II)-binding residues. Basal *O*-GlcNAc sites occur without any coexpression of OGT or OGT^mut^; persistent phosphorylation sites show high occupancy despite an increase in *O*-GlcNAcylation. An example of the PTM cross-talk on TET proteins is shown for TET3 Ser-360/Ser-361/Ser-362/Ser-368. *White arrowheads*, two co-occurring modifications; *black arrowheads*, three co-occurring modifications; *blunt arrows*, mutual exclusivity.

To date, the domains of TET proteins are largely uncharacterized, except for the conserved catalytic dioxygenase domain and the C*XX*C-type zinc finger at the N terminus of TET1 (8, 40, 48). Mapping the detected *O*-GlcNAc and phosphorylation sites to the TET protein sequence revealed that mostly the N terminus and low-complexity insert, which separates the two parts of the dioxygenase domain, were subjected to PTMs (Fig. 5). Remarkably, *O*-GlcNAcylation and phosphorylation rarely occurred at the exact same residue, although *O*-GlcNAcylation suppressed phosphorylation. Furthermore, the three TET proteins had different modification patterns: whereas TET1 was modified mostly at the N terminus and the very C-terminal part and was hardly glycosylated, TET2 and TET3 showed strong *O*-GlcNAcylation in the low-complexity insert region. The first 350 amino acids of TET3 remained free of PTMs. The observed pattern is not due to differences in sequence coverage, as the detected peptides are homogenously distributed over the whole protein sequence (supplemental Data S1).

Interestingly, some of the modifications were detected on the same peptides, indicating that they occurred together at the same molecule. For example, TET2 Ser-23 phosphorylation could be found with Ser-15 phosphorylation, and Ser-376 phosphorylation occurred only when Ser-374 was *O*-GlcNAcylated, but not when it was phosphorylated (Table 2). For TET3, a variety of PTM combinations could be observed for residues 360–368 and 1071–1077. Phosphorylation at Ser-362, for example, existed either alone or in combination with Ser-360 *O*-GlcNAcylation and Ser-368 phosphorylation. Phosphorylation of Ser-362 also co-occurred with *O*-GlcNAcylation of Ser-361. If Ser-362 was *O*-GlcNAcylated, however, no further modifications on this peptide were observed (Fig. 4 and Table 3). Apparently, some residues such as TET3 Ser-362 serve as *O*-GlcNAcylation/phosphorylation switches that can either promote or suppress neighboring PTMs. These data indicate a strong cross-talk between *O*-GlcNAcylation and phosphorylation at different residues. On the other hand, modifications on TET1 appeared more isolated, and no peptide bearing more than one modification was detected (Table 1). In summary, we detected many interdependent modification sites on TET proteins, suggesting that TET1, TET2, and TET3 are dynamically regulated by PTMs.

**TABLE 2:** **Detected modified peptides of TET2** (ph), phosphorylated; (*o*), *O*-GlcNAcylated; (ox), oxidized. Multiple modifications occurirng on one peptide are shown in boldface. Localization probability was calculated with the MaxQuant software (44). Residue numbering refers to the murine protein sequences specified in supplemental Data S1. The arithmetic mean ± S.D. of the occupancy is depicted for each data set. ND, not detected.

Modified amino acid	Localization probability	Modified sequence	Mean TET2	Mean TET2 + OGT^mut^	Mean TET2 + OGT
15	1.00	TTHAEGTRLS(ph)PFLIAPPS … K	0.57 ± 0.06	0.20 ± 0.01	0.01 ± 0.00
23	1.00	T … LS**(ph)**PFLIAPPS**(ph)**PIS … K	0.66 ± 0.09	0.32 ± 0.17	0.32 ± 0.04
39	0.98	LQNGS(ph)PLAERPHPEVNGDTK	0.45 ± 0.10	ND	ND
95	0.98	RT**(*o*)**VS**(*o*)**EPSLSGLHPNK	ND	0.06 ± 0.00	0.26 ± 0.17
97	1.00	TVS(ph)EPSLSGLHPNK	0.53 ± 0.26	0.49 ± 0.30	0.29 ± 0.02
97	0.97	RT**(*o*)**VS**(*o*)**EPSLSGLHPNK	ND	0.01 ± ND	0.29 ± 0.06
165	1.00	S … TSTTQESSGADAFPT(*o*)R	ND	0.74 ± 0.06	0.98 ± 0.02
317	0.98	SALDIGPS(*o*)RAENK	ND	ND	0.48 ± 0.03
374	0.82	DS(ph)ISPTTVTPPSQSLLAPR	ND	ND	0.34 ± 0.49
374	0.99	DS**(*o*)**IS**(ph)**PTTVTPPSQSLLAPR	ND	0.19 ± 0.22	0.49 ± 0.37
376	0.99	DS**(*o*)**IS**(ph)**PTTVTPPSQSLLAPR	1.00 ± ND	0.44 ± 0.38	0.30 ± 0.20
464	1.00	T … LPEQHQNDCGS**(ph)**PS**(ph)**PEK	0.79 ± 0.03	ND	ND
466	1.00	T … LPEQHQNDCGS**(ph)**PS**(ph)**PEK	0.79 ± 0.03	ND	ND
514	0.89	QT(*o*)QGSVQAAPGWIELK	ND	0.09 ± 0.03	0.59 ± 0.23
545	0.94	DIS(*o*)LHSVLHSQT … M(ox)SSK	ND	0.46 ± 0.10	0.78 ± 0.02
552	0.87	DIS**(*o*)**LHSVLHS**(*o*)**QT … MSSK	ND	0.13 ± ND	0.76 ± 0.20
561	0.95	DIS … VNQMS**(*o*)**S**(*o*)**K	ND	0.07 ± 0.00	0.79 ± 0.17
562	0.97	DIS … VNQMS**(*o*)**S**(*o*)**K*^a^*	0.01 ± 0.01	0.42 ± 0.06	0.80 ± 0.17
565	0.98	QS(*o*)TGNVNM(ox)PGGFQR	ND	ND	0.41 ± 0.04
603	1.00	AQMYQVQVNQGPS(ph)PG … K	0.41 ± 0.17	0.06 ± ND	0.14 ± 0.05
625	0.96	ALYQECIPRT(*o*)DPSS … R*^a^*	0.05 ± 0.01	0.73 ± 0.13	0.98 ± 0.02
746	0.98	VEESFCVGNQYS(*o*)K	ND	0.23 ± 0.16	0.83 ± 0.06
778	0.92	ILT(*o*)PNSSNLQILPSNDTHPACER	0.09 ± ND	0.31 ± 0.01	0.64 ± 0.05
807	1.00	EQALHPVGS(*o*)K	ND	0.01 ± ND	0.58 ± 0.12
889	1.00	ALPVPEQGGSQTQT(ph)PPQK*^b^*	0.57 ± 0.23	0.78 ± 0.30	0.57 ± 0.05
944	1.00	YPLS(ph)PPQENMSSR*^b^*	0.43 ± 0.14	0.46 ± 0.22	0.67 ± 0.11
951	0.97	PSSYRYPLSPPQENMS(ph)SR*^b^*	0.24 ± ND	0.32 ± ND	0.51 ± 0.68
1437	0.63	QM(ox)T(*o*)AQPQLS … R	ND	ND	0.67 ± 0.06
1443	0.98	QMTAQPQLS(*o*)GPVIR	ND	0.05 ± 0.05	0.52 ± 0.42
1613	0.87	D … PPIHT(*o*)LHQQTFGDSPSK	ND	ND	0.09 ± 0.10
1622	0.74	Y … TLHQQTFGDS(ph)PSK	0.45 ± 0.15	0.07 ± 0.04	0.76 ± ND
1640	0.76	DAFT(*o*)TNSTLKPN … K*^a^*	0.05 ± 0.01	0.50 ± 0.20	0.84 ± 0.03
1672	1.00	M(ox)DSHFM(ox)GAAS(*o*)R	ND	ND	0.93 ± 0.01
1749	1.00	TASAQELLYSLTGSS(ph)QEK	0.31 ± 0.02	0.07 ± 0.05	0.27 ± 0.02

*^a^* Basal *O*-GlcNAc sites.

*^b^* Persistent phosphorylation sites.

**TABLE 3:** **Detected modified peptides of TET3** (ph), phosphorylated; (*o*), *O*-GlcNAcylated; (ox), oxidized. Multiple modifications occurring on one peptide are shown in boldface. Localization probability was calculated with MaxQuant software (44). Residue numbering refers to the murine protein sequences specified in supplemental Data S1. The arithmetic mean ± S.D. of the occupancy is depicted for each data set. ND, not detected.

Modified amino acid	Localization probability	Modified sequence	Mean TET3	Mean TET3 + OGT^mut^	Mean TET3 + OGT
360	0.93	VEAPS**(*o*)**SS**(ph)**PAPVPS**(ph)**PISQR	ND	0.10 ± 0.07	0.91 ± 0.09
361	0.79	VEAPSS**(*o*)**S**(ph)**PAPVPSPISQR*^a^*	0.02 ± 0.01	0.52 ± 0.13	ND
362	1.00	VEAPSSS(ph)PAPVPSPISQR	0.03 ± 0.03	0.38 ± 0.03	0.01 ± ND
362	0.67	VEAPSSS(*o*)PAPVPSPISQR	ND	0.96 ± 0.02	ND
368	1.00	VEAPS**(*o*)**SS**(ph)**PAPVPS**(ph)**PISQR	0.34 ± ND	0.55 ± 0.40	0.28 ± ND
478	1.00	S(ph)RDM(ox)QPLFLPVR	0.46 ± 0.13	0.38 ± 0.13	0.66 ± ND
557	0.83	S(ph)PSPM(ox)VALQSGST … R	0.23 ± ND	0.22 ± 0.27	ND
557	0.76	S(*o*)PSPM(ox)VALQSGST … R	ND	ND	0.44 ± 0.30
1008	0.83	VS(*o*)SGAIQVLTAFPR	ND	0.91 ± 0.01	0.36 ± 0.51
1071	0.97	QEALELAGVT**(*o*)**T**(*o*)**DPGLSLK	ND	ND	0.96 ± 0.01
1072	0.89	QEALELAGVT**(*o*)**T**(*o*)**DPGLSLK	ND	ND	0.96 ± 0.01
1077	0.99	QEALELAGVTT**(*o*)**DPGLS**(*o*)**LK	ND	0.50 ± 0.40	0.53 ± 0.31
1105	0.89	YS(*o*)GNAVVESYSVLGS … R	ND	0.40 ± 0.07	0.73 ± 0.11
1252	0.94	VPQLHPAS(*o*)RDPSPFAQSSSCYNR	ND	0.42 ± ND	0.95 ± 0.03
1256	0.62	VPQLHPASRDPS(*o*)PFAQSSSCYNR*^a^*	0.04 ± 0.01	ND	0.98 ± 0.01
1263	0.84	VPQLHPASRDPSPFAQSSS(*o*)CYNR	ND	0.48 ± ND	0.98 ± 0.03
1282	0.88	QEPIDPLTQAES(*o*)IPR	ND	0.30 ± 0.06	0.91 ± 0.09
1293	1.00	T(*o*)PLPEAS … SGGPSMSPK*^a^*	0.01 ± 0.00	0.53 ± 0.06	0.99 ± 0.01
1318	1.00	TPLPEAS … SGGPSM(ox)S(ph)PK	0.43 ± 0.07	0.00 ± ND	ND
1351	0.61	LNSFGAS(ph)CLTPSHFPES … R	0.45 ± 0.39	ND	ND
1404	0.76	FGNGTSALTGPSLT(*o*)EK*^a^*	0.02 ± 0.02	ND	0.74 ± 0.15
1412	1.00	PWGM(ox)GT(*o*)GDFNPALK	ND	0.06 ± 0.00	0.65 ± 0.14
1651	0.72	Q … SAVT(*o*)VSSYAYTK	ND	ND	0.18 ± 0.05
1653	0.71	Q … SAVTVS(*o*)SYAYTK	ND	0.43 ± 0.19	0.73 ± 0.05
1654	0.77	Q … SAVTVS**(*o*)**S**(*o*)**YAYTK	ND	ND	0.24 ± 0.05
1658	0.99	G … TDSAVTVSSYAYT(*o*)K*^a^*	0.14 ± 0.03	0.12 ± 0.05	0.75 ± 0.14

*^a^* Basal *O*-GlcNAc sites.

## DISCUSSION

Because oxidation of 5-methylcytosine to 5-hydroxymethylcytosine, 5-formylcytosine, and 5-carboxylcytosine by TET proteins represents a potential mechanism for active DNA demethylation in higher vertebrates (3–5), these proteins are intensively investigated. Here, we provide evidence that all three TET proteins are subject to *O*-GlcNAcylation through OGT. This finding is in accordance with previous studies showing that TET1 and TET2 interact with OGT in embryonic stem cells and are *O*-GlcNAcylated (33, 34). TET3 has also been described to associate with OGT (32, 35) and to alter its subcellular localization dependent on glucose metabolism and *O*-GlcNAcylation (35). Not only does OGT directly modify TET proteins, but the interaction also promotes histone modifications such as H3K4me3 and H2BS112GlcNAc (31, 36). TET1 has been shown to associate with the repressive SIN3A complex (50), and TET2 and TET3 have been shown to associate with the SET1/COMPASS complex (31).

We have shown that, by default, TET proteins are phosphorylated. Basal *O*-GlcNAc levels are low but increase upon OGT expression. Simultaneously, the phosphorylation levels decrease. This finding identifies regulation of the phosphorylation signal as a novel function for TET *O*-GlcNAcylation. Interestingly, the underlying mechanism of this observation seems not to be direct competition for the serine or threonine residue that is to be modified, but rather proximal site competition as neighboring residues are interdependent (51). *O*-GlcNAcylation and phosphorylation of TET proteins occur at distinct amino acids, and several modifications of the same type often appear in close proximity in “modification islands,” *e.g. O*-GlcNAcylation at Ser-1252/Ser-1256/Ser-1263 of TET3 or phosphorylation at Ser-15/Ser-23/Ser-39 of TET2. It is important to note that only a few and more isolated *O*-GlcNAcylation sites are detected on TET1 compared with TET2 and TET3 and that glycosylation of TET1 is less conserved within biological replicates. We also did not observe *O*-GlcNAcylation of TET1 at Thr-535, which has been described previously as a major TET1 glycosylation site (33, 52). *O*-GlcNAcylation of TET1 seems to be very dynamic. This hypothesis is also supported by the fact that Myers *et al.* (52) detected TET1 Thr-535 *O*-GlcNAcylation in only one of three replicates, similar to our observation of heterogeneous TET1 glycosylation patterns.

To distinguish between mere interaction of TET proteins with OGT and catalytic activity of OGT on TET proteins, we used a catalytically inactive point mutant of OGT as a control. Interestingly, *O*-GlcNAcylation of TET proteins was slightly increased by OGT^mut^. This might be due to residual activity of the mutant (53) or, more likely, to recruitment of endogenous active OGT via trimerization of the tetratricopeptide repeat domain (54). Nevertheless, this supposed heterotrimer seems to target the same residues, as 91% of all detected *O*-GlcNAc sites in the OGT^mut^ samples were also modified in the OGT samples. Regarding phosphorylation, coexpression of OGT^mut^ also represents an intermediate state between the basal state, *i.e.* only TET expression, and coexpression of active OGT. 74% of all detected phosphorylation sites in the basal state also appeared during coexpression of OGT^mut^, arguing against steric hindrance of the kinase by the inactive enzyme as a mechanism for reduced phosphorylation.

In this study, we investigated TET protein PTMs dependent on OGT levels. The observed effect that *O*-GlcNAcylation of TET proteins reduces phosphorylation is of particular interest because protein *O*-GlcNAc levels are influenced by a variety of factors, such as different subcellular localization of OGT and nutrient availability, and seem to be tightly regulated (38). For example, *O*-GlcNAcylation of TET3 can be enhanced when cells are cultured in high-glucose medium, leading to nuclear export of TET3 (35). Furthermore, not only OGT activity but also OGT expression levels are tightly controlled in living cells. During chondrocyte differentiation, for example, OGT is up-regulated upon insulin stimulation (55). Moreover, the *Ogt* gene is located on the X chromosome and is subjected to dosage compensation through X chromosome inactivation (56). OGT-dependent dephosphorylation represents a novel mechanism by which TET proteins could be regulated in response to changing environmental conditions or during differentiation.

Interestingly, some residues remain stably phosphorylated even at high OGT levels. For TET2, they appear in close proximity to each other and just N-terminal of the cysteine-rich region. This persistence of phosphorylation suggests an important OGT-independent regulatory role of these residues that is of interest for future studies. Nevertheless, the majority of phosphorylation sites are reduced in occupancy upon *O*-GlcNAcylation. We thus observe two different types of phosphorylation: dependent on and independent of *O*-GlcNAcylation.

The hypothesis of interdependence of PTMs on TET proteins is further strengthened by the fact that some modifications are detected on the same peptides in stable combinations, whereas others occur as stand-alone modifications. Certain residues appear to be *O*-GlcNAcylation/phosphorylation switches that influence the PTM pattern on the neighboring amino acids. The observed cross-talk of modifications enables a variety of potential regulatory mechanisms that could fine-tune TET activity dependent on different environmental conditions such as nutrient availability.

To date, the domain architecture and three-dimensional structure of TET proteins are only poorly understood. The catalytic domain is highly conserved and homologous to other types of Fe(II)- and 2-oxoglutarate-dependent dioxygenases that act on nucleic acids (8, 57). Recently, the crystal structure of the catalytic region of TET2 has provided insights into the reaction mechanism (48). However, the large N terminus and low-complexity insert, which is characteristic for TET proteins, remain poorly understood in terms of both structure and function. So far, no homologous domains have been described, except for the C*XX*C-type zinc finger at the N terminus, and the insert region is predicted to be largely unstructured (8). In this study, we have shown that these two regions are subject to many dynamic PTMs. For TET1 and TET3, a few modification sites are also found at the very C terminus of the proteins, but the N terminus and insert region are the major targets of *O*-GlcNAcylation and phosphorylation. In general, the lower the conservation of one region, the more modification sites are detected. The selective modification of these regions might contribute to the regulation of TET protein activity, stability, or targeting. TET1, TET2, and TET3 have been described to co-localize with OGT at transcription start sites and influence gene expression (31, 34). The different modifications described in this study might alter binding of TET interaction partners and thus provide a possible explanation for the observed dual role in transcription activation and repression (58).

In summary, we have provided the first systematic mapping of *O*-GlcNAcylation and phosphorylation sites on TET proteins at amino acid resolution. The distribution of these PTMs and the described cross-talk provide new perspectives on the regulatory role of the so far poorly characterized non-catalytic domains: the N terminus and low-complexity insert region. The observed *O*-GlcNAcylation and phosphorylation are linked to metabolic conditions and thus provide a possible mechanism of TET protein regulation in response to external stimuli.

## Footnotes

4 The abbreviations used are:

mESC: mouse embryonic stem cell
OGT: *O*-linked GlcNAc transferase
PTM: post-translational modification.
